# A *Puccinia striiformis* f. sp. *tritici* Effector with DPBB Domain Suppresses Wheat Defense

**DOI:** 10.3390/plants14030435

**Published:** 2025-02-02

**Authors:** Raheel Asghar, Yu Cheng, Nan Wu, Mahinur S. Akkaya

**Affiliations:** School of Bioengineering, Dalian University of Technology, No. 2 Linggong Road, Dalian 116024, China; raheel@mail.dlut.edu.cn (R.A.); chengyu@nbu.edu.cn (Y.C.); wu_nan@mail.dlut.edu.cn (N.W.)

**Keywords:** double-psi beta-barrel (DPBB) fold, wheat, yellow rust disease, *Puccinia striiformis* f. sp. *tritici*, effector, plant immunity

## Abstract

Wheat (*Triticum aestivum* L.) is a primary crop globally. Among the numerous pathogens affecting wheat production, *Puccinia striiformis* f. sp. *tritici* (*Pst*) is a significant biotic stress agent and poses a major threat to world food security by causing stripe rust or yellow rust disease. Understanding the molecular basis of plant–pathogen interactions is crucial for developing new means of disease management. It is well established that the effector proteins play a pivotal role in pathogenesis. Therefore, studying effector proteins has become an important area of research in plant biology. Our previous work identified differentially expressed candidate secretory effector proteins of stripe rust based on transcriptome sequencing data from susceptible wheat (*Avocet S*) and resistant wheat (*Avocet YR10*) infected with *Pst*. Among the secreted effector proteins, *PSTG_14090* contained an ancient double-psi beta-barrel (DPBB) fold, which is conserved in the rare lipoprotein A (RlpA) superfamily. This study investigated the role of *PSTG_14090* in plant immune responses, which encodes a protein, here referred to as Pst-DPBB, having 131 amino acids with a predicted signal peptide (SP) of 19 amino acids at the N-terminal end, and the DNA sequence of this effector is highly conserved among different stripe rust races. *qRT-PCR* analysis indicated that expression levels are upregulated during the early stages of infection. Subcellular localization studies in *Nicotiana benthamiana* leaves and wheat protoplasts revealed that it is distributed in the cytoplasm, nucleus, and apoplast. We demonstrated that *Pst-DPBB* negatively regulates the immune response by functioning in various compartments of the plant cells. Based on Co-IP and structural predictions and putative interaction analyses by AlphaFold 3, we propose the probable biological function(s). Pst-DPBB behaves as a papain inhibitor of wheat cysteine protease; Pst-DPBB has high structural homology to kiwellin, which is known to interact with chorismate mutase, suggesting that Pst-DPBB inhibits the native function of the host chorismate mutase involved in salicylic acid synthesis. The DPBB fold is also known to interact with DNA and RNA, which may suggest its possible role in regulating the host gene expression.

## 1. Introduction

Wheat (*Triticum aestivum* L.) is a critical food security crop and a primary source of nutrition for humans globally. However, wheat production is significantly impacted by various abiotic and biotic stresses, notably from pathogenic microorganisms. Among these pathogens, wheat stripe rust, caused by *Puccinia striiformis* f. sp. *tritici* (*Pst*), is an airborne disease and one of the most severe threats to crop yield. Stripe rust, also called yellow rust, is an ancient affliction that periodically resurfaces in various regions due to the pathogen’s expanding geographical range and the emergence of new strains capable of overcoming resistance in cultivars in use. The pathogen’s ability to disperse over considerable distances enables it to incite large-scale epidemics within short timeframes. Environmental conditions, such as high humidity and low temperatures, exacerbate the infection, leading to rapid disease progression and substantial crop loss [1,2,3,4].

Plant pathogens generally proliferate within the extracellular and intracellular space after entering through stomata, water pores, or mechanical wounds [5]. Fungi, after invasion, extend their hyphae across the surface of plant cells to develop haustoria for absorbing nutrients [6]. The interfaces between the haustorial plasma membrane, the extracellular matrix, and the host plasma membrane are critical sites and routes of secretion of effectors into the subcellular environment of the host [7,8], while plants utilize innate immune responses and systemic signals from infection sites to counteract these invasions [9].

Pathogens encounter two layers of plant defense after overcoming pre-existing barriers [10]. The first layer involves recognizing pathogen-derived molecules, termed pathogen-associated molecular patterns (PAMPs), which initiate host resistance via specific receptors, an immune mechanism known as PAMP-triggered immunity (PTI) [11,12]. Pathogens may disrupt PTI by secreting effectors that modulate host metabolic pathways and defense responses, leading to effector-triggered susceptibility (ETS) [13]. Conversely, the recognition of an effector by host resistance proteins (R proteins) activates a second layer of defense, termed effector-triggered immunity (ETI), often characterized by hypersensitive responses that induce localized cell death [9] by the recognition of a class of conserved receptors expressed intracellularly, categorized under nucleotide-binding leucine-rich repeat (NB-LRR) proteins [14]. These receptors exhibit high specificity in effector recognition, either directly or indirectly binding to effectors to induce ETI. Accessory proteins may serve as targets for pathogen virulence or mimic these targets [15]. Responses associated with PTI and ETI exhibit similarities but may differ in intensity [16]. Concurrently, natural selection promotes the evolution of novel resistance genes, enabling plants to mount renewed ETI responses.

Given the pivotal role of effector proteins in mediating plant–pathogen interactions, our aim focuses on understanding the function and molecular mechanisms of candidate effector proteins. Our previous transcriptomic analyses identified *Pst* candidate effector PSTG_14090, which is highly expressed during the early stages of infection [17]. This effector exhibits a conserved structural feature known as the double-psi beta-barrel (DPBB) fold, henceforth referred to as Pst-DPBB. The DPBB fold is conserved across various enzymes in fundamental biochemical processes, such as formate dehydrogenase (PDB ID 1FDO) [18], AMP phosphorylase (PDB ID 4GA6) [19], phosphate propanoyltransferase (PDB ID 5CUO) [20], valosin-containing protein (VCP)-like ATPase (PDB ID 5G4F) [21], DNA polymerase (PDB ID 5IJL) [22], and RNA polymerase (PDB ID 6ASG) [23]. The DPBB fold has also been identified in several plant pathogen effectors, as recent research suggests that DPBB domain-containing kiwellin-like effectors are widely distributed across eukaryotic organisms, including plant pathogens and non-pathogens [24].

One notable effector containing the DPBB fold is PNPi, a *Pst* effector that interacts with the wheat NPR1 protein via its C-terminal DPBB domain to compete with NPR1’s interaction with transcription factor TGA2.2 in the nucleus [25]. Later, PNPi was also validated to interact with wheat pathogenesis-related TaPR1a in the apoplastic space [26]. Therefore, PNPi can attenuate multiple defense responses to pathogens by targeting different components in wheat plants. Another effector containing the DPBB fold from *Rhizoctonia solani* is named RsRlpA, and its gene was highly induced during the early-stage infection of sugar beet seedlings. RsRlpA suppresses the hypersensitive response (HR) induced by the Avr4/Cf4 complex in transgenic *Nicotiana benthamiana* plants and functions as a protease inhibitor, suppressing the reactive oxygen species (ROS) burst [27]. Another DPBB domain-containing apoplastic effector, MoRlpA, has been identified in the rice blast fungus *Magnaporthe oryzae*. While MoRlpA was characterized through an in silico approach, this study highlighted its structural potential to interact with the rice cathepsin B protease OsCathB, which is secreted during infection and plays a crucial role in programmed cell death (PCD) and hypersensitive responses. MoRlpA acts as a protease inhibitor, suppressing OsCathB activity in the apoplast to interfere with PTI and ETI [28].

Although PNPi has been thoroughly studied as a DPBB domain-containing effector in *Pst*, the DPBB fold is located in the C-terminal, covering one-third of its sequence excluding the signal peptide (SP). This structural difference highlights the unique potential of Pst-DPBB to act as a model for understanding the role of DPBB-containing effectors in *Pst* infection. For PNPi, its ability to suppress plant immune responses may rely on functions encoded by the other two-thirds of its sequence, rather than exclusively reflecting the activities of the DPBB fold. Conversely, Pst-DPBB provides a more focused opportunity to explore the contributions of the DPBB fold itself in effector functions. We aimed to elucidate the functional significance of Pst-DPBB in modulating wheat immune responses and to uncover its potential interaction with host proteins, contributing to a broader understanding of effector biology. By integrating structural, functional, and interaction analyses, we hypothesize that Pst-DPBB functions as a negative regulator of plant immunity, potentially targeting key host defense pathways. These findings will contribute to a deeper understanding of effector biology and the molecular mechanisms underlying plant–pathogen interactions.

## 2. Results

### 2.1. Confirmation and the Characterization of Pst-DPBB as a Secreted Effector

**Figure 1:**
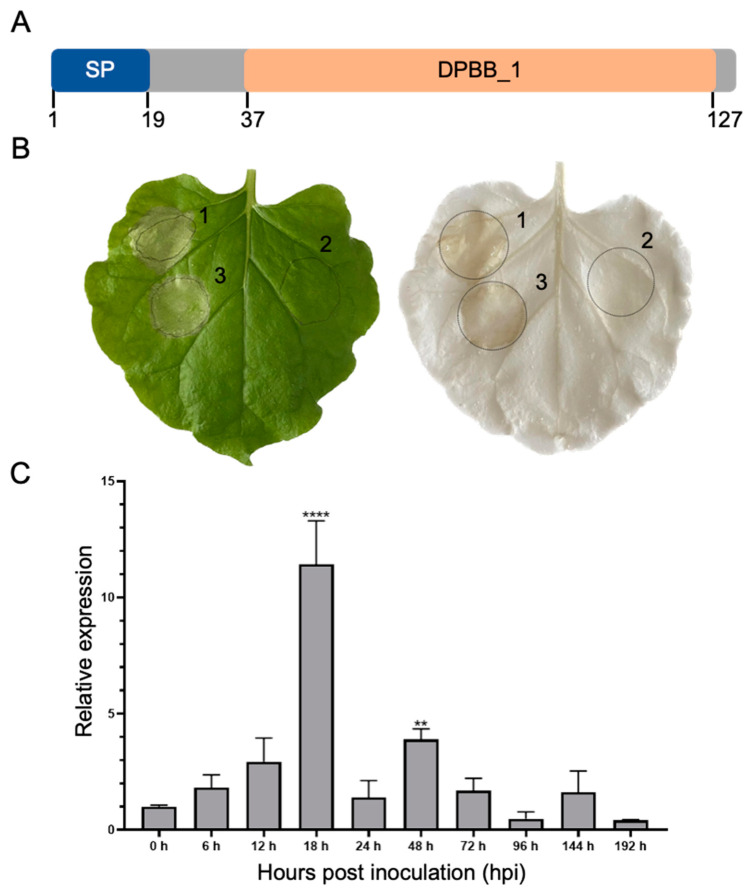
Features of Pst-DPBB: validation of signal peptide, and transcript levels of Pst-DPBB at various stages following *Pst* inoculation. (**A**) SignalP 6.0 and InterProScan were conducted to identify signal peptide (SP) and the presence of a conserved domain, respectively. (**B**) Secretory function determination of the predicted signal peptide of Pst-DPBB. (1) pTRBO-Pst-SP-SCR1 (full-length SCR1 with native signal peptide), (2) pTRBO-Pst-SCR1^ΔSP^ (full-length SCR1 without native signal peptide), and (3) pTRBO-SP^Pst-DPBB^-SCR1^ΔSP^ (Pst-DPBB signal peptide fused to Pst-SCR1^ΔSP^) were transiently expressed in *N. benthamiana* by *Agrobacterium* GV3101 infiltration. Phenotypes were observed after 7 days post-infiltration (left panel). Cell death is further detected after decolorization (right panel). (**C**) Expression levels of Pst-DPBB. The relative transcription levels of Pst-DPBB were analyzed using the comparative threshold method (2^−ΔΔCt^), comparing levels just before inoculation to the control and normalizing to the expression level of *PstEF-1α* as the reference gene. Means and standard deviations are derived from three independent biological replicates, with significance between different time points and the control analyzed using a one-way ANOVA test (** indicates *p* < 0.01, **** indicates *p* < 0.0001).

The Pst-DPBB protein is small in size with 131 amino acids (aa), containing 6 cysteines, and the first 19 aa of the N-terminal is a predicted SP (Figure 1A), which are the key features of pathogen effectors. To verify the secretion of Pst-DPBB, we swapped the SP of Pst-DPBB with a known effector protein PstSCR1. PstSCR1 is secreted to apoplast to induce cell death [29]. The transient expression of the PstSCR1 with the fused SP of Pst-DPBB instead of its native SP in tobacco showed cell death, as expected with extracellularly functioning native PstSCR1, which verifies the prediction of the signal peptide region on the N-terminus and confirms Pst-DPBB is a typical secreted effector protein (Figure 1B).

The transcript levels of Pst-DPBB were measured at different infection stages ranging from 0 to 192 h post-inoculation (hpi) (Figure 1C). The expression was detected as highly upregulated in infected wheat leaves at 18 and 48 hpi compared to 0 hpi, reaching a peak at 18 hpi coinciding with the initiation of *Pst* haustoria formation in wheat (Figure 1C), suggesting it plays a role in the early stage of infection.

The coding sequence (CDS) of Pst-DPBB (KNE92489.1) in different *Pst* races, namely PST-78, CYR32, PST-38S102, PST-Yr9, PST-K, PST-11-281, and PST-93-210, showed a single base difference in the nucleotide at position 288 among the different races (Figure S1); however, this variation does not change the amino acid sequence, indicating that Pst-DPBB is highly conserved among *Pst* races.

### 2.2. The Pst-DPBB Is Associated with Diverse Functional Roles

**Figure 2:**
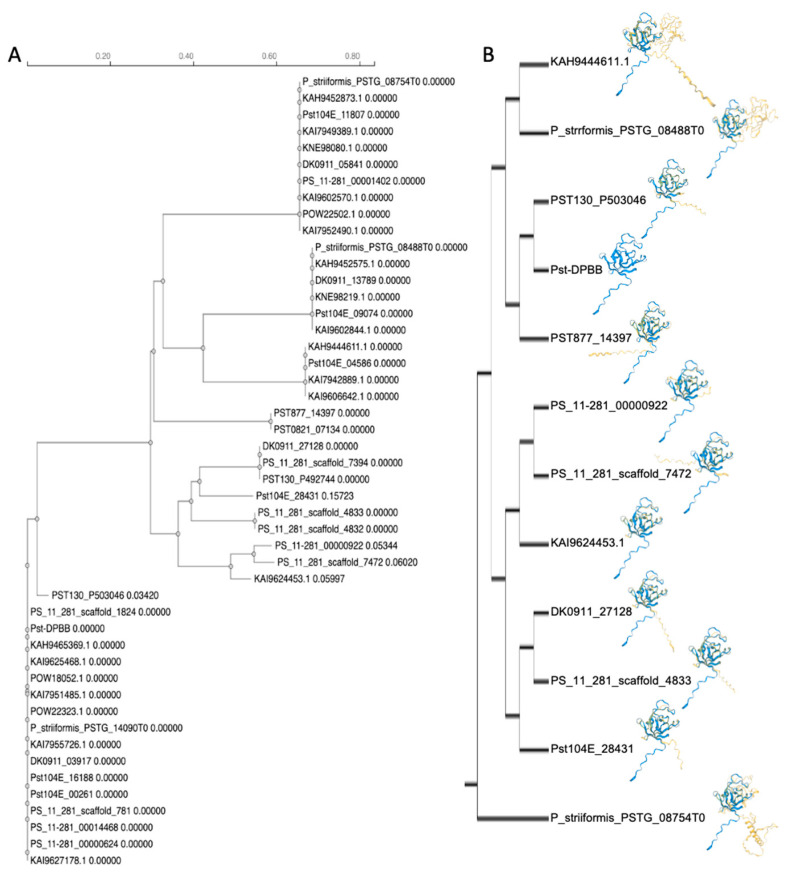
Sequence and structural similarities of Pst-DPBB-like proteins without the signal peptide. (**A**) Sequence-based phylogenetic tree of Pst-DPBB-like proteins constructed with Clustal Omega. (**B**) Structural phylogenetic tree generated using Foldseek, highlighting the alignment of Pst-DPBB with identical and homologous proteins from various races and species. The protein IDs are the same as the IDs of the proteome data [30]. The structure in blue is Pst-DPBB, and the aligned structures are in yellow.

The structure of the Pst-DPBB effector protein was predicted using AlphaFold 3 (AF3), yielding a predicted template modeling (pTM) score of 0.82, resulting in a high-confidence model indicating a highly reliable structural prediction. The template modeling score is the evaluation of resemblance or the degree of structural alignment between proteins; values closer to 1.0 indicate higher structural similarity and values above 0.5 are considered reliable matches. The predicted structure of Pst-DPBB was compared to 8102 effector proteins identified in a previous study obtained by the search of the secretome of twelve *Pst* races and two other *Puccinia striiformis* species [30]. Among these, 256 proteins were found to be structurally similar to Pst-DPBB. A structural phylogenic tree using the alignment obtained by FoldMason [31] along with Pst-DPBB resulted in 34 most closely related protein structures (Table S1). Searching the entirety of the proteome of the *Pst* races and two other *Puccinia striiformis* species [30] led to the determination of 14 additional proteins having identical amino acid sequences as Pst-DPBB. The sequence-based alignment using Clustal Omega [32] resulted in homologous and the same sequence proteins (Figure 2A). We picked a representative protein with the same amino acid sequence for a structure-based similarity search. The structure of PST130_P503046 was determined as the closest to Pst-DPBB. The proteins have the following annotations in the PDB database, i.e, the cellulose-binding protein (4l48_C, 4jcw_A), papain inhibitor (5ntb_B), Expansin-YoaJ (4fer_A), EXLX1 (7wvr_A, cellulose-binding protein), and ripening-related protein 3 (6fpg_H) (Figure 2B). Both analyses consistently showed that Pst-DPBB is part of a highly conserved group of DPBB domain-containing proteins. This combined approach reveals the evolutionary relationships of DPBB domains and their conserved structural features.

**Figure 3:**
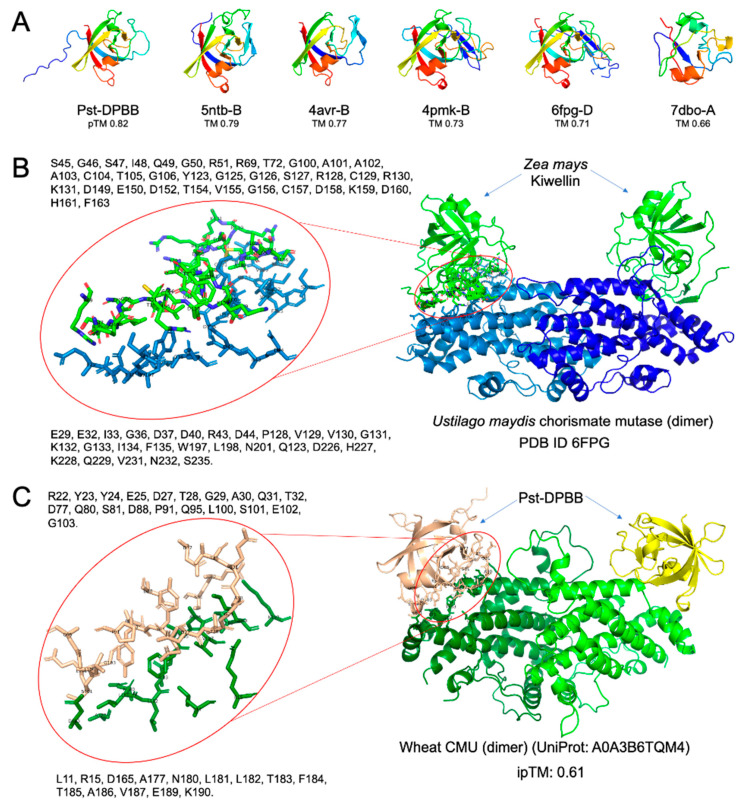
Structural homology analysis using Foldseek compared to Pst-DPBB structure. (**A**) The predicted structure of Pst-DPBB by AF3 (pTM 0.82). 5ntb-B, a papain inhibitor from *Streptomyces mobaraensis*; 4avr-B, a hypothetical protein Pa4485 from *Pseudomonas aeruginosa*; 4pmk-B, a kiwellin protein from *Actinidia chinensis*; 6fpg-D, a kiwellin protein from *Zea mays*; and 7dbo-A, a DPBB domain from a VCP-like ATPase in *Thermoplasma acidophilum*. (**B**) The interaction between *Zea mays* kiwellin and *Ustilago maydis* chorismate mutase (PDB ID: 6FPG). (**C**) The interaction interface of Pst-DPBB with wheat chorismate mutase (UniProt accession: A0A3B6TQM4), predicted by AF3.

When the predicted structure of the Pst-DPBB effector protein was compared to the Protein Data Bank (PDB) database that Foldseek maintains [33], 232 structural matches were found in the PDB database (Table S2). Among the analyzed matches, 58 had a TM score ≥ 0.5, suggesting reliable structural alignments, while the remaining 174 scored below this threshold, with weaker structural resemblance. The top matches, all having a DPBB domain (Figure 3A), are a papain inhibitor [34]; a hypothetical protein Pa4485 (RlpA-like) [35]; a kiwellin protein from two different species [36,37]; and a DPBB domain of a VCP-like ATPase [38].

Recently, kiwellin-like fold-containing effectors (KLEs) were shown as candidate secretory effector proteins of plant pathogens and rust fungi by comparative structural genomics [24]. The kiwellin protein was first discovered in kiwi plant as an allergen having a DPBB domain [39]; later, kiwelin-like proteins were determined in various plant species [24,36]. The KLEs of rust are more similar to plant kiwellin-like fold-containing proteins, having N-terminal intrinsic disordered regions (IDRs) and a core DPBB domain, which is known to bind to carbohydrates in various other DPBB fold-containing proteins, such as expansins [24]. Interestingly, in *Ustilago maydis,* an effector protein was identified as chorismate mutase 1 (CMU1), and experimentally, it was shown to form a complex with a particular *Zea mays* (*Z. mays*) kiwellin protein that binds to the active site of CMU1 with high specificity, but not many other kiwellin-like proteins in maize [36,40]. The crystal structure of this interaction, as shown in Figure 3B (PDB ID: 6FPG), demonstrates how the kiwellin protein specifically binds CMU1, potentially interfering with its enzymatic function.

Based on this, we compared the AF3-generated structural model of Pst-DPBB, identified as a kiwellin-like effector (KLE), to a wheat CMU (Figure 3C). The model, with an ipTM (interface-predicted template modeling) score of 0.61, suggests a moderately confident prediction of this binding mode, revealing a strikingly similar interaction depicted in PDB ID: 6FPG (Figure 3B). This resemblance highlights a conserved interaction mechanism, where Pst-DPBB KLE appears to mimic the structural features of kiwellin to bind to its host target, wheat CMU. Such binding could inhibit the enzyme’s native function in the defense-related synthesis of salicylic acid [41,42], potentially modulating its activity and influencing host metabolic pathways to facilitate pathogen virulence.

### 2.3. Pst-DPBB Is Localized to the Cytoplasm, Nucleus, and Apoplast

**Figure 4:**
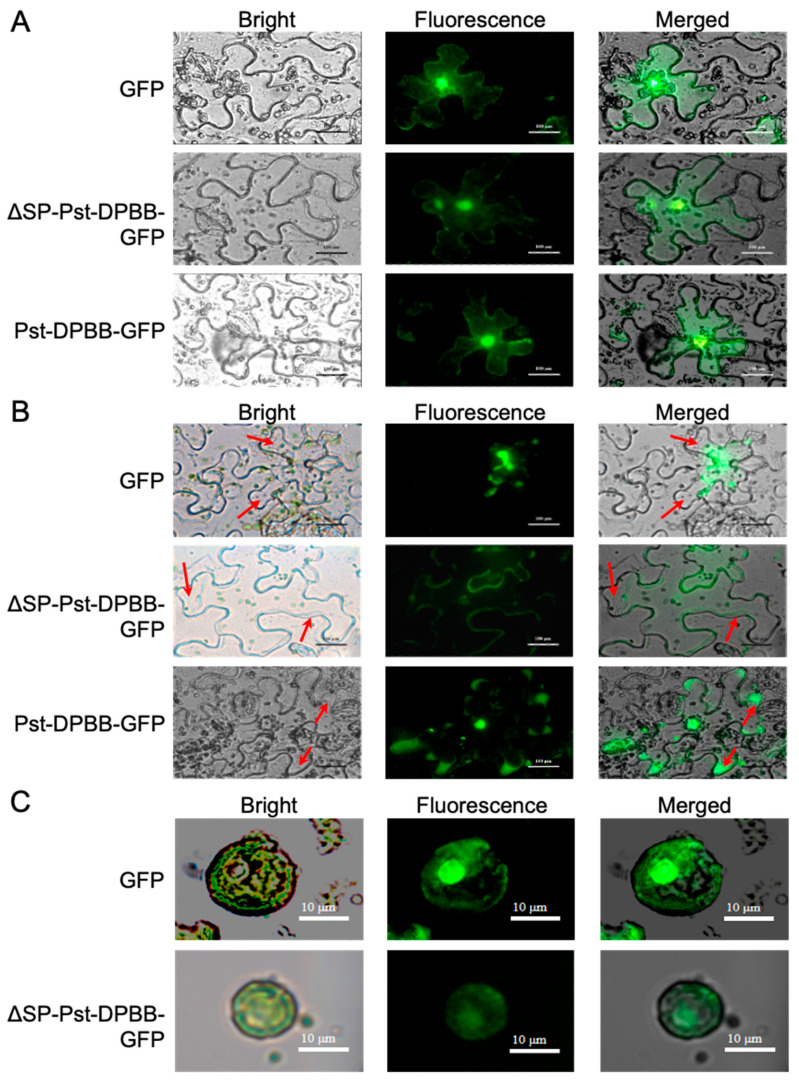
Subcellular localization of Pst-DPBB in plant cells. *N. benthamiana* epidermal cells transiently expressing pTRBO-ΔSP-Pst-DPBB-GFP, pTRBO-SP-Pst-DPBB-GFP, and pTRBO-GFP (control) were observed at 5 days post-infiltration (dpi) without treatment (**A**) and after plasmolysis (**B**). Additionally, pTRBO-ΔSP-Pst-DPBB-GFP and pTRBO-GFP (control) were transiently expressed in wheat protoplasts (**C**). Observations were made using a fluorescence microscope with an excitation wavelength filter of 465–495 nm and an emission wavelength filter of 512–558 nm. The red arrows in (**B**) indicate the apoplastic space formed after plasmolysis. Scale bars represent 100 μm for images of *N. benthamiana* epidermal cells and 10 μm for images of wheat protoplasts.

The Pst-DPBB effector protein was transiently expressed as a GFP fusion, both with and without its signal peptide (SP), in *N. benthamiana* epidermal cells and wheat mesophyll protoplasts (Figure 4). In *N. benthamiana* leaves, constructs lacking the SP (ΔSP-Pst-DPBB-GFP) showed GFP fluorescence localized in the cytoplasm and nucleus, similar to the GFP control (Figure 4A). After plasmolysis, GFP fluorescence for SP-Pst-DPBB-GFP (with SP intact) was observed in the apoplastic space, indicating the secretion of Pst-DPBB to the cell surface (Figure 4B). In wheat protoplasts, ΔSP-Pst-DPBB-GFP again showed fluorescence in the cytoplasm and nucleus, consistent with its localization in *N. benthamiana* cells and similar to the GFP control (Figure 4C). These findings suggest that Pst-DPBB can localize to the cytoplasm, nucleus, and apoplast, indicating its potential involvement in multiple biological processes in various compartments during infection.

### 2.4. Pst-DPBB Negatively Regulates Plant Immune Response

**Figure 5:**
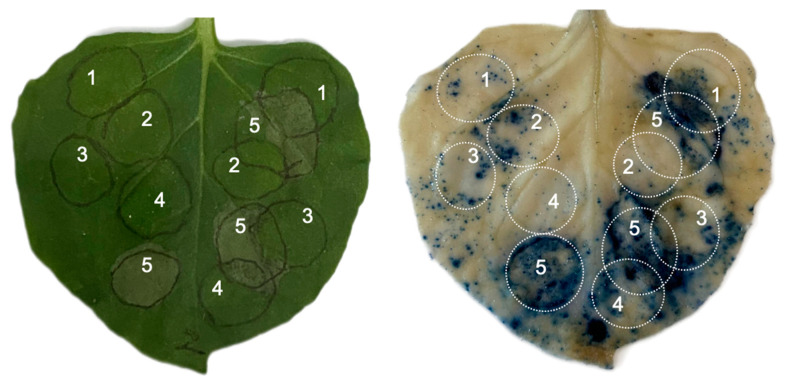
Suppression of INF1-triggered cell death by Pst-DPBB in *N. benthamiana*. The constructs (1) pTRBO-GFP (negative control), (2) pGR106-Avr3a^KI^ (positive control), (3) pTRBO-SP-Pst-DPBB, (4) pTRBO-ΔSP-Pst-DPBB, and (5) pGR106-INF1 were transiently expressed or co-expressed in *N. benthamiana* via agroinfiltration. The leaf necrosis phenotype was photographed at 5 days post-infiltration (dpi). The right panel displays the same leaf as the left panel after staining with 0.25 mg/mL trypan blue.

Oomycete elicitor INF1 can trigger widespread immune responses and cause cell death in tobacco; however, some effectors such as avirulent effector protein Avr3a^KI^ from *Phytophthora infestans* inhibit INF1-induced cell death [43]. The results, as shown in Figure 5, indicated that INF1 expression alone induced programmed cell death (PCD) as expected. Notably, when INF1 was co-expressed with Pst-DPBB possessing SP, the PCD induced by INF1 was suppressed, which is consistent with the positive control where Avr3a^KI^ was able to inhibit the PCD caused by INF1, and the effector protein lacking SP is ineffective in the suppression of INF1-mediated cell death. Based on these data, we propose that Pst-DPBB on the cell surface negatively regulates plant immune response.

**Figure 6:**
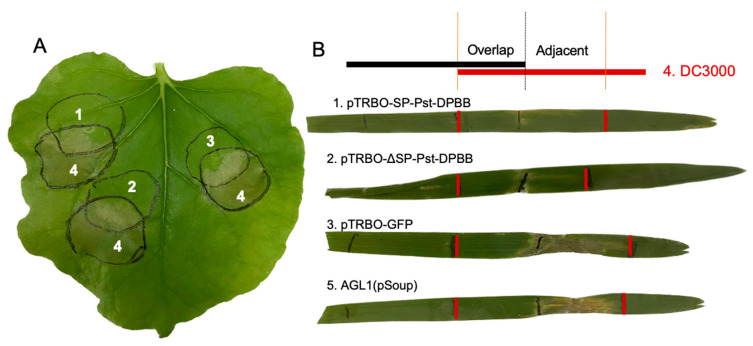
*Pst-DPBB* inhibits programmed cell death (PCD) triggered by *Pseudomonas syringae.* DC3000 in barley, but not in tobacco. (**A**) The constructs; (1) pTRBO-SP-Pst-DPBB, (2) pTRBO-ΔSP-Pst-DPBB, and (3) pTRBO-GFP (control) were transiently co-expressed with (4). *Pseudomonas syringae* DC3000 in *N. benthamiana*. The leaf necrosis phenotype was evaluated at 3 dpi. (**B**) Suppression of DC3000-triggered cell death by Pst-DPBB in barley. *Agrobacterium* AGL1 (pSoup) carrying (1) pTRBO-SP-Pst-DPBB, (2) pTRBO-ΔSP-Pst-DPBB, (3) and pTRBO-GFP (control), and (5) AGL1 (pSoup) cells alone were infiltrated into the first leaf of 10-day-old barley (left half, marked with black brackets). Two days later, (4) DC3000 was infiltrated into the right border of the pre-infiltration area (marked with red brackets), creating a partially overlapping region. Leaf necrosis phenotypes were observed after 5 days.

The PTI response of the effector was also tested against the *Pseudomonas syringae* pv. *tomato* strain DC3000, which is pathogenic to *Arabidopsis thaliana* and tomato, and triggers a hypersensitive response (HR) in non-host plants such as *N. benthamiana* and barley [44,45]. Interestingly, the expression of Pst-DPBB with or without SP and the GFP control showed similar levels of necrosis on *N. benthamiana* leaves, suggesting that Pst-DPBB cannot inhibit DC3000-induced cell death in tobacco (Figure 6A). Nevertheless, the influence of Pst-DPBB in barley was different from that in tobacco. Using the *Agrobacterium* AGL1 (pSoup)-mediated transient expression system in barley, AGL1 cells transformed with Pst-DPBB constructs (with or without SP) and GFP (control) were infiltrated into barley leaves; following two days, DC3000 cells were infiltrated into the overlapping areas. The responses were detected after 5 days (Figure 6B) as the suppression of cell death in the overlapping regions of infiltration sites. The fact that the AGL1 (pSoup) transformants or non-transformants suppress cell death is an indication of the non-pathogenicity of *Agrobacterium* AGL1 (pSoup), triggering PTI and limiting the release of DC3000 Type III secretion effectors within the infection areas. Therefore, if the same area of barley leaves had been pre-infiltrated with non-pathogenic AGL1 (pSoup), the HR associated with DC3000-induced ETI would have been suppressed. However, in the adjacent areas outside of the overlapping region (the far-right infection area), the infiltration of AGL1 (pSoup) and GFP expression no longer inhibited DC3000-induced PCD, whereas the expression of Pst-DPBB with or without SP still inhibited PCD. This indicates that the overexpression of the effector protein Pst-DPBB can suppress DC3000-induced defense responses in barley, and this suppression can extend to adjacent areas.

### 2.5. Pst-DPBB Suppresses PTI-Related Callose Deposition in Barley

**Figure 7:**
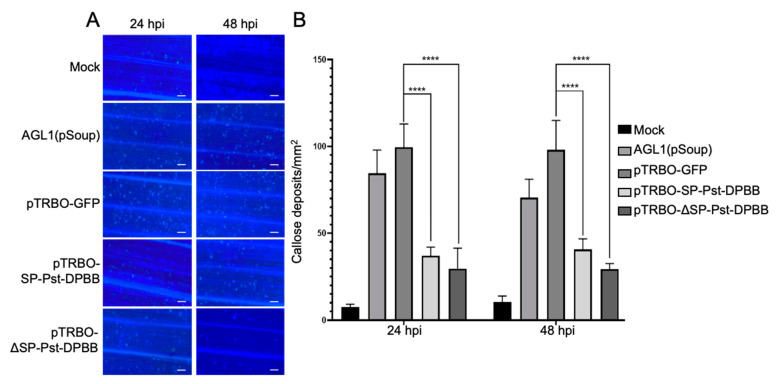
Pst-DPBB inhibits PTI-associated callose deposition. (**A**) Samples were collected from barley leaves at 24 and 48 h after infiltration with either infiltration buffer (Mock), AGL1 (pSoup), and AGL1 (pSoup) carrying pTRBO-GFP, pTRBO-SP-Pst-DPBB, and pTRBO-ΔSP-Pst-DPBB. The samples were examined using a fluorescence microscope following 0.05% aniline blue staining. Scale bars = 100 μm. (**B**) The average number of callose deposits per mm^2^ in barley leaves infiltrated with infiltration buffer (Mock), AGL1 (pSoup), and AGL1 (pSoup) carrying pTRBO-GFP, pTRBO-SP-Pst-DPBB, and pTRBO-ΔSP-Pst-DPBB at 24 or 48 hpi. Means and standard deviations were calculated from three independent biological replicates. **** indicate a significant difference (*p* < 0.0001) compared to the pTRBO-GFP sample, as determined by two-way ANOVA.

Callose is one of the components of plant cell walls, playing an important role in plant immunity. Callose deposition is a fundamental response, as it strengthens the plant cell wall, enhancing the plant’s resistance to pathogens. During pathogen invasion, plants, by recognizing PAMPs, trigger immunity. We investigated the effect of the overexpressing effector protein on callose deposition in barley to study the role of Pst-DPBB in plant PTI responses (Figure 7A). Similar to the AGL1 strain of *Agrobacterium* as a positive control, Pst-DPBB, when overexpressed with or without SP, significantly inhibited callose deposition by approximately 60–70% compared to the pTRBO-GFP control at both 24 and 48 h post-infiltration (hpi) (Figure 7B), indicating that it suppresses the PTI response in plants.

### 2.6. Pst-DPBB Influences Expression of PTI-Associated Marker Genes in Tobacco

**Figure 8:**
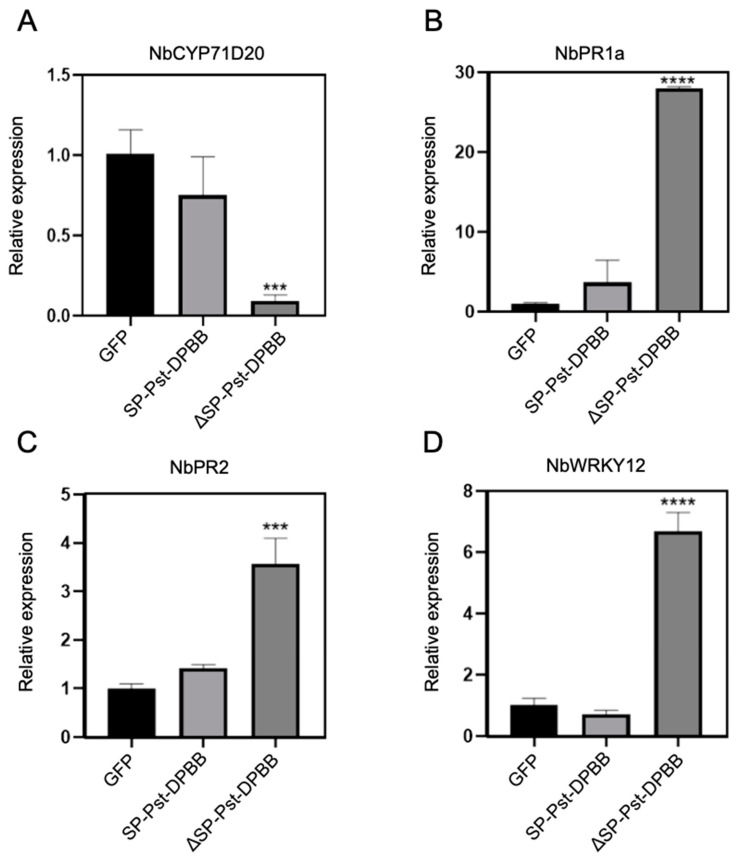
Expression analysis of PTI-related marker genes following the overexpression of *Pst-DPBB* in *N. benthamiana*. The constructs pTRBO-GFP (control), pTRBO-SP-Pst-DPBB, and pTRBO-ΔSP-Pst-DPBB were transiently expressed in *N. benthamiana* via agroinfiltration. After 24 h, 20 μM flg22 was infiltrated into the same region. The transcription levels of defense-related genes, including *NbCYP71D20* (**A**), *NbPR1a* (**B**), *NbPR2* (**C**), and *NbWRKY12* (**D**), were measured at 12 h post-infiltration using quantitative reverse transcription PCR (qRT-PCR). *NbActin* served as the internal reference gene for *NbCYP71D20* and *NbPR1a*, while *NbEF1α* was used as the reference gene for *NbPR2* and *NbWRKY12*. Means and standard deviations were calculated from three independent biological replicates, and significance between different samples and the control was assessed using a one-way ANOVA test (*** indicates *p* < 0.001, **** indicates *p* < 0.0001).

To elucidate the negative regulatory role of the effector protein Pst-DPBB on the plant PTI response, we tested the expression levels of flg22-mediated PTI markers, *NbCYP71D20*, *NbPR1a*, *NbPR2*, and *NbWRKY12*, in *N. benthamiana* following the transient overexpression of Pst-DPBB. A day after flg22 pre-treatment, the plants were infiltrated with Pst-DPBB constructs (pTRBO-ΔSP-Pst-DPBB, pTRBO-SP-Pst-DPBB) and the control (pTRBO-GFP). After 12 hpi, qPCR was performed to measure the expression levels of the PTI-related marker genes. The results demonstrated that, compared to the GFP control, the expression level of *NbCYP71D20* decreased by 90% in leaves, with the transient expression of Pst-DPBB lacking the SP. Meanwhile, *NbPR1a*, *NbPR2*, and *NbWRKY12* expression levels increased by 28, 3.6, and 8 times, respectively (Figure 8). The transient expression of Pst-DPBB with intact SP had no significant impact on the expression of the four PTI-related marker genes. Based on these data, the effector protein Pst-DPBB partially inhibits the flg22-induced PTI response, likely by inhibiting one or a few of the defense responses induced by flg22.

### 2.7. Silencing of Pst-DPBB Reduces Pst Pathogenicity

**Figure 9:**
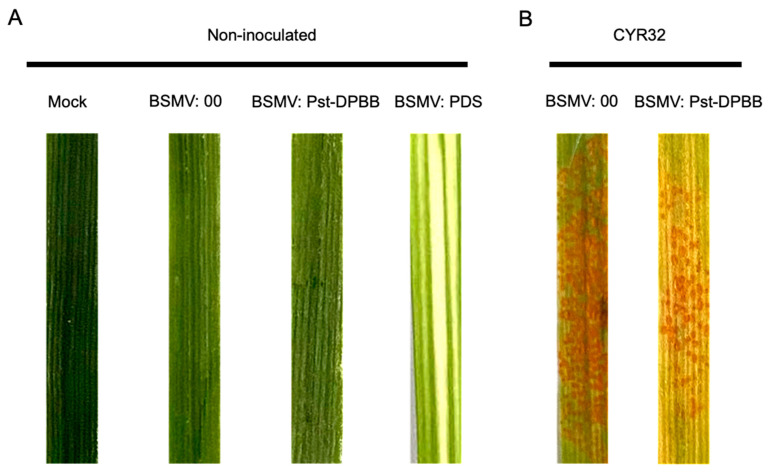
BSMV-mediated HIGS of *Pst-DPBB* reduces the pathogenicity of *Pst*. (**A**) Phenotypes of the third leaves of wheat plants treated with tobacco sap containing BSMV after two weeks of growth. “Mock” indicates wheat leaves inoculated with PBS buffer, “BSMV:00” represents wheat leaves inoculated with the BSMV empty vector pCaBS-γb, “BSMV: Pst-DPBB” indicates wheat leaves with *Pst-DPBB* gene silencing, and “BSMV: PDS” represents wheat leaves with phytoene dehydrogenase (PDS) silencing. (**B**) Observed disease response in *Pst-DPBB* silenced plants inoculated with the virulent *Pst* race CYR32 at 14 dpi. The third leaves of wheat plants inoculated with the BSMV empty vector and BSMV: Pst-DPBB were inoculated with *Pst* race CYR32 at 10 days post-BSMV treatment.

To further characterize the function of Pst-DPBB in *Pst* pathogenicity, we knocked down the expression of *Pst-DPBB* in wheat–*Pst* interactions using barley stripe mosaic virus (BSMV)-mediated host-induced gene silencing (HIGS). A specific silencing fragment of *Pst-DPBB* was designed to generate the recombinant virus constructs, and the BSMV: γ*-TaPDS* construct was used as a virus indicator. At 10 days post-BSMV inoculation, BSMV: γ-*TaPDS*-inoculated wheat leaves exhibited photobleaching, confirming successful virus system activity (Figure 9A). The third leaves of BSMV-inoculated wheat plants were then inoculated with the virulent *Pst* race CYR32, which is pathogenic to control wheat plants. Disease phenotypes were observed at 14 days post-inoculation (dpi). Wheat leaves inoculated with the BSMV: γ-empty vector showed typical susceptibility to *Pst* race CYR32, with abundant spore formation. In contrast, BSMV: Pst-DPBB-silenced wheat leaves exhibited reduced spore formation (Figure 9B). These data indicate that knocking down the expression of *Pst-DPBB* weakened *Pst* pathogenicity, highlighting the role of Pst-DPBB in stripe rust fungal virulence.

### 2.8. Candidate Interactors of Pst-DPBB in Planta

The screening of target proteins for the effector protein Pst-DPBB was conducted using co-immunoprecipitation and mass spectrometry (CoIP-MS/MS). *Agrobacterium* strains carrying the plasmids pTRBO-GFP (control) and pTRBO-ΔSP-Pst-DPBB-GFP were infiltrated into 6-week-old *N. benthamiana* plants. After 5 days post-infiltration (dpi), total protein was extracted from the tobacco leaves. GFP-Trap Agarose was used to isolate the expressed proteins, and the GFP, ΔSP-Pst-DPBB-GFP, and their interacting proteins were subsequently eluted. The expression of both GFP and ΔSP-Pst-DPBB-GFP proteins in the tobacco was confirmed via SDS-PAGE and Coomassie brilliant blue staining (Figure S2A), followed by Western blot analysis (Figure S2B). In the ΔSP-Pst-DPBB-GFP eluted sample, an additional GFP band of approximately 26.87 kDa was observed during GFP immunoprecipitation. This band may be attributed to the cleavage of the ΔSP-Pst-DPBB-GFP fusion protein during expression or purification processes. The successful elution of both GFP and ΔSP-Pst-DPBB-GFP from the GFP-Trap indicated an effective co-immunoprecipitation of the target proteins.

The eluted protein samples were sent for mass spectrometry analysis to identify potential interacting targets. A total of 42,592 spectra were obtained for the control group (GFP). After search engine identification, 741 spectra were matched, leading to the identification of 91 proteins and 193 peptide sequences. For Pst-DPBB, 43,157 spectra were obtained, with 3326 spectra matched, resulting in the identification of 824 proteins (53 of which were also identified in the GFP control) and 2433 peptide sequences. The proteins identified in the control were excluded, leaving the interacting proteins that specifically bind to the Pst-DPBB-GFP protein via GFP-Trap agarose beads. Gene Ontology (GO) and KOG (Eukaryotic Orthologous Groups) annotation analyses were performed, selecting proteins involved in immune system processes and defense mechanisms as predicted interaction targets of the effector protein. The predicted interaction targets of Pst-DPBB are displayed in Table 1.

**Figure 10:**
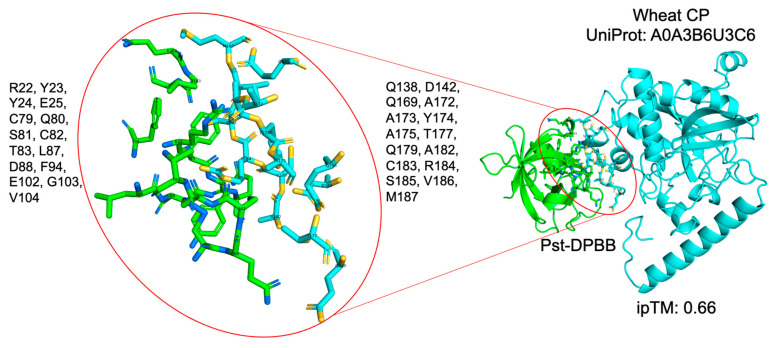
Predicted interaction interface between Pst-DPBB and the wheat cysteine proteinase using AF3. The putatively interacting amino acids of Pst-DPBB (green) and the wheat cysteine proteinase (cyan) are presented in Figure 10.

While our CoIP-MS/MS experiments have identified several candidate interactors involved in immune system processes and defense mechanisms defined by GO terms, structural modeling using AlphaFold3 (AF3) yielded low ipTM scores. This discrepancy may arise due to the candidates being a non-host plant (*Nicotiana benthamiana*) and the limitations in computational modeling, particularly the absence of key cofactors or third-party proteins that might facilitate or stabilize these interactions in vivo but are not included in the AF3 models. Weak or transient interactions, which are often biologically significant, may also be underrepresented in AF3 predictions. Despite the low ipTM scores, the biological relevance of these experimentally validated interactors remains consistent with the functional roles of Pst-DPBB’s structural homologs, such as the papain inhibitor. We noticed that the Co-IP experiment determined two protease enzymes and thus focused on determining sequence homologs of these particular proteinases in wheat and conducting AF3. Notably, a wheat cysteine proteinase with the UniProt accession number A0A3B6U3C6 was identified with 30% sequence similarity to cysteine proteinase 3 of tobacco as a potential interactor of Pst-DPBB (Figure 10). To further explore the evolutionary conservation of this interaction, we aligned the wheat cysteine proteinase with *N. benthamiana* cysteine proteinase 3, identified through Co-IP MS/MS experiments. The sequence alignment revealed a 30% sequence identity between the two proteinases, highlighting conserved regions that may underlie their functional similarity (Figure S3).

## 3. Discussion

Effector proteins are secreted by pathogens to regulate host physiology and innate immunity. A comprehensive understanding of the types of effector proteins, their transport within host cells, and their functions has become a frontier in effector biology research. This study focused on the wheat stripe rust fungus candidate effector protein Pst-DPBB, employing a combination of bioinformatics analysis and experimental approaches to preliminarily explore its function and potential regulatory mechanisms in plant immunity.

The effector protein Pst-DPBB encodes a total of 131 amino acids, with an N-terminal signal peptide spanning 19 amino acids. This signal peptide was experimentally confirmed to possess secretion functionality, aligning with the fundamental characteristics of effector proteins, which are generally low in molecular weight and exhibit secretion capability. Unlike commonly rapidly evolving effector proteins, the sequence alignment results of Pst-DPBB show high conservation within the species of *Puccinia striiformis*, which may indicate its significant role in the successful evolution of this group of pathogens, and the limited structural changes of this protein are likely under evolutionary constraints [46,47,48]. Secondly, by expressing the signal peptide-containing SP-Pst-DPBB-GFP and the signal peptide-deleted ΔSP-Pst-DPBB-GFP fusion proteins in *N. benthamiana* and wheat protoplasts, it was observed that fluorescent signals could be detected in the apoplast, cytoplasm, and nucleus, indicating that Pst-DPBB targets all these subcellular locations within the host cell and exerts variable functions, potentially participating in multiple biological processes. Therefore, we investigated, in addition to their role in plant immunity, potential host-interacting proteins experimentally, together with 3D structural analyses, which served in predictions of the roles of the Pst-DPBB effecter in multiple compartments. The proteins we identified as structurally homologous display names such as papain inhibitor, kiwellin, RlpA-like, expansin, cellulose-binding protein, and ripening-related protein. Reference to the proteins is used interchangeably in different databases. A double-psi beta-barrel (DPBB) fold is common to all the proteins. One superfamily is called the rare lipoprotein A (RlpA) [49]. Some proteins within the RlpA superfamily have enzymatic activity, such as a protein identified as RlpA in *Escherichia coli*, which functions as a lytic transglycosylase [50]; this activity targets cell wall polysaccharides under particular physiological conditions for degradation, similar to the membrane-associated lytic transglycosylase A (MltA) of *E. coli* and *Neisseria gonorrhoeae* [51].

Another set of proteins is expansins; they mediate cell wall loosening non-enzymatically and are present in all plants and some microbial organisms. In plants, they play roles from germination to fruit ripening, including abiotic and biotic stresses [52]. Expansin-related proteins (ERPs) as a group of microbial proteins are documented and shown to induce plant defense immunity as PAMPs [53]. Pst-DPBB, having only a core DPBB domain and a short protruding N-terminal part, does not possess a transglycosylase active site. Nevertheless, its DPBB domain may bind to carbohydrates as in expansin-like proteins [54].

Structural similarity also hits kiwellin proteins from plants such as *Actinidia chinensis* (kiwi) and Zea mays. Maize-encoded kiwellin specifically blocks the catalytic activity of the chorismate mutase (CMU1) from the fungal pathogen *Ustilago maydis* by hindering substrate access to the active site of CMU1, and then disarms the metabolic activity to reprogram plant metabolism and immune responses [36]. Reversely, a recent study revealed that kiwellin protein-like fold-containing effectors (KLEs) are extensively present in different rust fungi. One of the KLEs, Pstr_13960, from the Indian Pst race Yr9, suppressed the BAX-induced cell death. Interestingly, it was shown targeting the chloroplasts in *Nicotiana benthamiana*. Its interaction with plant chorismate mutases was presented by molecular docking [35]. It can be assumed that Pst-DPBB may act as KLEs, probably interacting with the wheat chorismate mutases reducing the SA-mediated defense, since two molecules of the effector form a complex to the dimerized cytoplasmic wheat CMU based on AF3 with a high ipTM score, validating the observation of the effector in cytoplasm.

The predicted structure of Pst-DPBB showed high similarity to the identified papain inhibitor in *Streptomyces mobaraensis*, suggesting that Pst-DPBB may function as a papain-like cysteine protease (PLCP) inhibitor in wheat. Plant proteases are known to play significant roles in plant immunity [55], and aim to degrade secreted pathogen effectors mostly on the cell surface; in turn, pathogen effectors inhibit protease activities [56,57,58]. MoErs1, a species-specific effector protein secreted by *Magnaporthe oryzae*, functions as a protease inhibitor directly interacting with and inhibiting the activity of OsRD21, a PLCP known to be involved in rice immunity. This was demonstrated through yeast two-hybrid (Y2H) and co-immunoprecipitation (Co-IP) assays, protease activity assays confirming inhibition, and virulence assays showing reduced pathogenicity in MoERS1 knockout mutants [59]. Interestingly, when CoIP-MS/MS was employed to screen for several proteins involved in immune system processes and defense mechanisms that could be potential interaction targets of Pst-DPBB, we found one candidate target to be papain-like cysteine protease 6. Based on the structural similarity between Pst-DPBB and the *Streptomyces* protease inhibitor (SPI), it is speculated that Pst-DPBB may exhibit papain-like protease inhibitory activity. The identification of papain and other proteinases as candidate interactors in Table 1 further supports the functional similarity between Pst-DPBB and the papain inhibitor. These findings highlight potential pathways through which Pst-DPBB may contribute to wheat stripe rust pathogenesis.

The predicted interactions of Pst-DPBB with wheat chorismate mutase and cysteine proteinase suggest it may help the pathogen by interfering with important plant processes. Pst-DPBB’s interaction with chorismate mutase (Figure 3C) could disrupt the pathways needed for plant defense, while its interaction with cysteine proteinase (Figure 10) might block proteins involved in the plant’s immune response. Using a known interaction between kiwellin and chorismate mutase as a reference (Figure 3B), these results suggest that Pst-DPBB targets multiple host proteins to weaken the plant’s defenses.

Interestingly, DPBB domains are also part of some RNA- and DNA-binding proteins, suggesting a broader functional versatility. These domains are implicated in processes such as nucleic acid recognition and stabilization, often vital for regulating gene expression and replication [37,60,61]. The presence of DPBB in Pst-DPBB might hint at additional roles beyond protein–protein interactions, including targeting nucleic acid-associated processes in the host. Further studies are needed to explore whether Pst-DPBB exploits its DPBB domain to interfere with host transcriptional regulation or other nucleic acid-related pathways, adding another layer to its potential as a multifunctional effector.

Our CoIP-MS/MS experiments identified several other candidate interactors of Pst-DPBB, including proteases and immunity-related proteins, providing strong experimental evidence for their relevance. However, the structural modeling of some interactors with Pst-DPBB within *Nicotiana benthamiana* using AF3 resulted in low ipTM scores, indicating low confidence in the computational predictions of interaction. This low confidence may reflect limitations of computational tools such as AF3, which do not account for key biological factors, such as the requirement of cofactors or third-party proteins that might stabilize these interactions in vivo. Additionally, weak or transient protein–protein interactions, which are common in biological systems, are challenging to model accurately. Despite these limitations, the functional roles of the identified interactors align well with those of Pst-DPBB’s structural homologs, such as the papain inhibitor.

Most importantly, as shown by various experiments, this *Pst* effector triggers plant immunity as PTI. Co-expressing Pst-DPBB and INF1 in tobacco revealed that the signal peptide-containing Pst-DPBB can inhibit INF1-induced PCD. In contrast, the signal peptide-deleted effector protein cannot. Since INF1 is an elicitor targeting the plant apoplast, it is inferred that Pst-DPBB must be secreted into the plant apoplast to inhibit INF1-induced defense responses. In contrast to the ability to suppress INF1-induced cell death in tobacco, Pst-DPBB does not inhibit PCD induced by *Pseudomonas syringae* DC3000 in tobacco, but can inhibit DC3000-induced death in barley. This discrepancy may be due to different mechanisms by which Pst-DPBB responds to plant defense in tobacco and barley. Additionally, the overexpression of Pst-DPBB in barley effectively reduces the accumulation of callose in barley leaves. To clarify the role of the effector protein Pst-DPBB in the plant PTI response, the effect of Pst-DPBB on the expression of four PTI-related marker genes induced by flg22 was examined, revealing that Pst-DPBB only partially suppresses the expression of *NbCYP71D20*, indicating that Pst-DPBB has a limited inhibitory effect on the flg22-induced PTI response. Overall, Pst-DPBB has a function in negatively regulating plant immune responses.

## 4. Materials and Methods

### 4.1. Plant Materials, Fungal Isolate, and Bacterial Strains

The susceptible wheat (*Triticum aestivum* L.) cultivar MingXian169 (MX169), along with tobacco (*N. benthamiana*) and barley (*Hordeum vulgare*), was used in this study. All plants were grown in a chamber with 16 h of light and 8 h of darkness. The light intensity was set at 200 μmol·m^−2^·s, 80% humidity, 18 °C for wheat and barley, and 23 °C for tobacco. Chinese yellow rust race CYR32 is used for wheat inoculations. Spores were amplified on MX169, when the seedlings reached the two-leaf stage. We used freshly collected spores for experimental purposes. For transient expressions, *Agrobacterium tumefaciens* (*A. tumefaciens*) strain GV3101 and AGL1 (pSoup) were cultured in Luria–Bertani (LB) medium at 28 °C for tobacco and barley, respectively [62].

### 4.2. Plasmid Constructs

The CDS of *Pst-DPBB* (GenBank #KNE92489.1) was synthesized and cloned into the vector pGGC000 (Addgene plasmid #48858) by GenScript (Nanjing, China). The recombinant plasmid was named pGGC000-SP-*Pst-DPBB* and served as the source of the CDS fragment for further constructions. The transient expressions of *Pst-DPBB* were conducted without a signal peptide in tobacco, wheat protoplasts, and barley after cloning into pJL-TRBO-G (Addgene plasmid #80083) at the N-terminus of GFP to form recombinant plasmid pTRBO-ΔSP-*Pst-DPBB*-GFP by GenScript (Nanjing, China). The full length of *Pst-DPBB* was cloned into pJL-TRBO-G using pGGC000-SP-*Pst-DPBB* as a template for amplifying the CDS to form the recombinant plasmid pTRBO-SP-*Pst-DPBB*-GFP using a seamless cloning kit (Beyotime#D7010s, Shanghai, China) and used in tobacco transient gene expressions. To validate the functionality of the *Pst-DPBB* signal peptide, the 57 bp corresponding to the predicted signal peptide from *Pst-DPBB* was assembled with the linearized pTRBO-*Pst*-SCR1^ΔSP^ vector [18] to form the recombinant plasmid pTRBO-*Pst-DPBB*-SP-SCR1^ΔSP^ using a seamless cloning kit (Beyotime#D7010s, Shanghai, China). For BSMV-mediated host induced gene silencing (HIGS) of *Pst-DPBB*, a 210 bp fragment of *Pst-DPBB* was amplified and inserted into the linearized pCaBS-γb vector (digested by *Apa* I) to create the plasmid pCaBS-γb-*Pst-DPBB* [63]. The primers used for all constructs are listed in Table S3.

### 4.3. Signal Peptide Prediction and Validation

The signal peptide sequence of *Pst-DPBB* was predicted using SignalP version 6.0 (https://services.healthtech.dtu.dk/service.php?SignalP, accessed on 20 May 2023) [64]. Constructs of pTRBO-*Pst*-SP-SCR1, pTRBO-*Pst*-SCR1^ΔSP^, and pTRBO-*Pst-DPBB-*SP-SCR1^ΔSP^ were introduced into *A. tumefaciens* strain GV3101 via heat shock [65], and positive transformants were selected using kanamycin and rifampicin. The individual clones were verified by PCR. For tobacco leaf infiltration, recombinant *A. tumefaciens* strains were cultured in LB medium for 48 h, harvested, washed three times with infiltration buffer (10 mM MES, 10 mM MgCl_2_, 0.1 mM acetosyringone, pH 5.7), and resuspended in the same buffer to a final 0.6 of OD_600nm_. The cells were incubated at room temperature for 2 h before infiltration. The cell suspensions were infiltrated into tobacco leaves using a needleless syringe [66]. The symptoms were observed and photographed at 7 days post-infiltration (dpi) on the leaves and after the decolorization in the ethanol and acetic acid (1:1 *v*/*v*) mixture.

### 4.4. Domain Prediction, Sequence Alignment, Structural Prediction, and Phylogenetic Analysis of Pst-DPBB Protein

Domain analysis was carried out using InterProScan (https://www.ebi.ac.uk/interpro/, accessed on 20 May 2022) [67] to identify conserved domains and predict potential functional sites within the protein sequences. Sequence alignment was conducted using BlastN (https://blast.ncbi.nlm.nih.gov/Blast.cgi?PROGRAM=blastn, accessed on 10 November 2022) to compare target sequences against *Puccinia Striiformis* in the NCBI database. DNAMAN software (version 9.0, Lynnon Biosoft, San Ramon, CA, USA) was used to align the CDS sequences.

The structure of the Pst-DPBB protein was predicted using AlphaFold 3 (https://golgi.sandbox.google.com/, accessed on 7 November 2024) [68]. This model was then analyzed using Foldseek (https://search.foldseek.com/search, accessed on 8 November 2024), which compared the Pst-DPBB structure against known proteins in public databases, helping to identify structural similarities and infer potential functions [33]. The Pst-DPBB with wheat chorismate mutase (A0A3B6TQM4) and wheat cysteine proteinase (A0A3B6U3C6) interaction was modeled using AlphaFold3 (AF3) (https://golgi.sandbox.google.com/, accessed on 7 November 2024 and 10 January 2025, respectively).

The predicted structure of Pst-DPBB was searched against a dataset of 8102 effector proteins identified in a previous study [30]. From this dataset, 256 proteins were identified as homologs of Pst-DPBB. These 256 proteins, along with Pst-DPBB, were aligned using FoldMason (https://search.foldseek.com/foldmason accessed on 28 November 2024) to construct a structural phylogenetic tree, revealing a clade of 34 closely related structures, including Pst-DPBB [31]. To refine this dataset further, the entire sequence of Pst-DPBB was searched against the proteomes of 14 *Puccinia striiformis* races analyzed in our previous study, which led to the identification of 14 additional proteins. These newly identified proteins were integrated into the initial dataset, resulting in a total of 48 proteins that were then aligned using Foldseek for structural analysis. Additionally, a sequence-based phylogenetic tree was constructed using Clustal Omega (https://www.ebi.ac.uk/jdispatcher/msa/clustalo accessed on 28 November 2024) to explore the evolutionary relationships among these proteins [32].

### 4.5. Transient Expression Assays for Subcellular Localization in Planta

Constructs of pTRBO-GFP, pTRBO-ΔSP-*Pst-DPBB*-GFP, and pTRBO-SP-*Pst-DPBB*-GFP were introduced into *A. tumefaciens* strain GV3101 via heat shock, and positive transformants were selected using kanamycin and rifampicin. The individual clones were verified by PCR. For infiltration into tobacco leaves, the recombinant strains of *A. tumefaciens* were grown in LB medium for 48 h, harvested, washed three times with infiltration buffer, resuspended in the buffer to a final 0.6 value of OD_600nm_, and incubated at room temperature for 2 h before infiltration. Cell suspensions were infiltrated into tobacco as above. The subcellular localization of *Pst-DPBB* in the epidermal cells of tobacco leaves was observed at 5 dpi using a fluorescence microscope (Nikon Ni-U, Tokyo, Japan) with an excitation wavelength filter of 465–495 nm and an emission wavelength filter of 512–558 nm. For plasmolysis, tobacco leaves were treated with 5% NaCl for 10 min before observation. For wheat protoplast preparation, 2 g of two-leaf-stage MX169 wheat leaves were cut into small pieces and soaked in 20 mL of 0.4 M mannitol for 10 min. The pieces were then transferred into 10 mL of enzyme solution (1.5% cellulose, 0.75% macerozyme, 10 mM MES, 1 mM CaCl_2_, 0.1% BSA, 0.6 M mannitol) under vacuum for 30 min in the dark, then returned to normal pressure and shaken at 70 rpm for 1.5 h in the dark. The protoplast suspension was filtered through a 75 μm nylon mesh to remove undigested tissue after adding 10 mL of W5 solution (154 mM NaCl, 125 mM CaCl_2_, 5 mM KCl, 2 mM MES, pH 5.7). The filtered protoplasts were centrifuged at 100× *g* for 5 min at room temperature to collect purified protoplasts. The wheat protoplasts were diluted to a concentration of 1×10⁶ cells/mL using MMG solution (4 mM MES, 0.6 M mannitol, 15 mM MgCl_2_, pH 5.7). For transformation, 10 μL of 2 μg/μL plasmid pTRBO-ΔSP-*Pst-DPBB*-GFP or pTRBO-GFP was added to 100 μL of protoplasts. Then, 110 μL of PEG-Ca^2+^ solution (0.2 M mannitol, 100 mM CaCl_2_, 50% PEG-4000) was added, and the mixture was incubated in the dark at room temperature for 20 min. To stop the transformation, 440 μL of W5 solution was added, and the protoplasts were centrifuged at 200× *g* for 5 min at room temperature. The protoplasts were resuspended in 100 μL of WI solution (4 mM MES, 0.5 M mannitol, 5 mM KCl), incubated in the dark at room temperature for 24 h [69]. Observations for localization was conducted on a fluorescence microscope (Nikon Ni-U, Japan).

### 4.6. Total RNA Extraction and qPCR

Wheat leaves were collected at different time points of inoculation with fresh CYR32 spores: 0, 6, 12, 18, 24, 48, 72, 96, 144, and 192 hpi (hours post-inoculation). Each time point had three biological replicates, and the samples were placed in 1.5 mL RNase-free tubes, frozen in liquid nitrogen, and stored at −80 °C. For tobacco infiltration, *A. tumefaciens* strain GV3101 carrying pTRBO-ΔSP-*Pst-DPBB*-GFP or pTRBO-SP-*Pst-DPBB*-GFP was cultured, adjusted to 0.6 OD_600nm_, and injected into 6-week-old tobacco leaves. After 24 h, 20 μM flg22 was injected into the same spot. The leaves were harvested at 12 hpi, frozen in liquid nitrogen, and stored at −80 °C. Total RNA was extracted from the frozen samples using TRIzol reagent (Invitrogen #15596026CN, Carlsbad, CA, USA) according to the manufacturer’s protocol, which included homogenizing the tissue, separating phases with chloroform, and purifying the RNA. RNA concentrations were adjusted to 500 ng/μL. Genomic DNA was removed, and first-strand cDNA synthesis was carried out using the SweScript RT II First Strand cDNA Synthesis Kit (Servicebio #G3333, Wuhan, China). The reverse transcription reactions of 20 μL were set up, containing the genome removal mix, primers, and SweScript RT II enzyme mix, followed by incubation at 25 °C for 5 min, 55 °C for 15 min, and 85 °C for 5 s. The resulting cDNA was stored at −20 °C. For qRT-PCR, a 15 μL reaction mixture was prepared, including 1 μL cDNA, 7.5 μL SYBR Green master mix (Servicebio #G3326, Wuhan, China), 0.3 μL forward primer, 0.3 μL reverse primer, and 5.9 μL water. The PCR was run on a CFX Connect Real-Time PCR Detection System (Bio-Rad, Hercules, CA, USA), with 45 cycles of denaturation at 95 °C for 15 s, and annealing and extension at 60 °C for 30 s. A melting curve was generated to check the specificity of the amplification. Data analysis was conducted using CFX Maestro v2.2, with the relative gene expression calculated by the 2^−ΔΔCt^ method [70]. Each test was repeated five times. The primers used for qRT-PCR are listed in Table S3.

### 4.7. Programmed Cell Death Assays

To assess the role of *Pst-DPBB* on INF1-induced programmed cell death (PCD) in tobacco leaves, recombinant strains of *A. tumefaciens* GV3101 harboring pTRBO-ΔSP-*Pst-DPBB*-GFP or pTRBO-SP-*Pst-DPBB*-GFP were cultured and adjusted to 1.0 OD_600nm_ in infiltration buffer. On the following day, after 24 h, GV3101 harboring INF1 (0.1 OD_600nm_) was infiltrated at the same sites of the samples [71]. pTRBO-GFP was used as the negative control, while pGR106-Avr3a^KI^ was used as the positive control [43]. The response was photographed after 5 d. The leaves were then soaked in trypan blue staining solution (1 mg/mL trypan blue, 20% lactic acid, 20% glycerol, 20% phenol) overnight, followed by treatment with a decolorizing solution (glycerol–acetic acid–ethanol = 1:1:3) and photographed. To test the role of *Pst-DPBB* on *Pseudomonas syringae* pv. *tomato* DC3000-induced PCD on tobacco leaves, the recombinant strains of *A. tumefaciens* GV3101 harboring pTRBO-ΔSP-*Pst-DPBB*-GFP or pTRBO-SP-*Pst-DPBB*-GFP were cultured and adjusted to 1.0 OD_600nm_ with infiltration buffer. After a day, DC3000 (0.02 OD_600nm_) was injected at the same sites as the samples were infiltrated. The pTRBO-GFP construct was used as a negative control. The response was photographed after 2 days. For assessing the role of *Pst-DPBB* on DC3000-induced PCD on barley leaves [72], the recombinant strains of *A. tumefaciens* AGL1 (pSoup) harboring pTRBO-ΔSP-*Pst-DPBB*-GFP or pTRBO-SP-*Pst-DPBB*-GFP were cultured and adjusted to an OD_600nm_ of 1.0 with infiltration buffer, followed by infiltration into 10-day-old barley leaves [73]. After 2 days, DC3000 (0.1 OD_600nm_) was injected at the same positions as the samples, and the pTRBO-GFP construct was used as a negative control. The response on the leaves was photographed after 5 days.

### 4.8. Callose Deposition Assay

Recombinant strains of *A. tumefaciens* AGL1 (pSoup) harboring pTRBO-ΔSP-*Pst-DPBB*-GFP or pTRBO-SP-*Pst-DPBB*-GFP were cultured, adjusted to 1.0 OD_600nm_ with infiltration buffer and infiltrated into 10-day-old barley leaves. At 24 and 48 h post infiltration (hpi), the collected barley leaves were soaked in a solution of ethanol and acetic acid (1:1 *v*/*v*) for 1 day until they became transparent. Following this, the leaves were cleaned with 50% ethanol for 15 min, rinsed with ddH_2_O for 10 min, treated with 0.5 M NaOH for 10 min, and rinsed again with ddH_2_O for 10 min. The leaves were then soaked in 67 mM K_2_HPO_4_ for 1 h prior to staining with 0.05% aniline blue in 67 mM K_2_HPO_4_ overnight. Finally, the stained leaves were observed under a fluorescence microscope (Nikon Ni-U, Japan) with an excitation wavelength filter of 361–389 nm and an emission wavelength filter of 430–490 nm, and the number of callose depositions was counted in one mm^2^ area using ImageJ software (https://fiji.sc/).

### 4.9. BSMV-Mediated Host Induced Gene Silencing of Pst-DPBB

The *Agrobacterium* strains carrying pCaBS-α, pCaBS-β, and pCaBS-γb-*Pst-DPBB* were cultured and adjusted to an OD_600nm_ of 0.7 before being mixed in equal proportions. This mixture was kept in the dark at 28 °C for 3 h and then injected into *N. benthamiana* leaves. The infiltration buffer solution was used as the blank control, pCaBS-γb was used as the negative control, and pCaBS-γb-*TaPDS* was used as the positive control for virus-induced gene silencing. After 12 d, two injected leaves were collected, ground in PBS buffer, and the leaf sap was filtered through a 75 μm nylon membrane. Subsequently, 1% celite was added to the sap and mixed. The tobacco sap was applied to 2-leaf-stage MX169 wheat leaves by rub-inoculation [63]. After 10 d, the third leaves were further inoculated with fresh CYR32 spores. Then, 14 days post-*Pst* inoculation, the phenotypes of the third leaves were photographed.

### 4.10. Screening of Pst-DPBB Interacting Proteins in Planta

Recombinant strains of *A. tumefaciens* GV3101 harboring pTRBO-ΔSP-*Pst-DPBB-GFP or* pTRBO-GFP (control) were cultured and adjusted to an OD_600nm_ of 1.0 with infiltration buffer before being infiltrated into tobacco leaves. After 5 d, the leaves exhibiting GFP signals were collected, ground in protein extraction buffer (50 mM HEPES, 0.15 M KCl, 1 mM EDTA, 0.5% Triton X-100, 1 mM DTT, 10 mM NaF), and centrifuged at 13,000 rpm at 4 °C for 15 min to collect the supernatant of crude protein. The protein was then immunoprecipitated using GFP-TRAP (Proteintech Cat #gta, Wuhan, China) according to the manufacturer’s protocol. To verify that the eluted protein was the target protein, protein electrophoresis and immunoblotting were conducted. Protein electrophoresis was performed using the Bolt™ 12% Bis-Tris Mini Protein Gel (Invitrogen, #NW00122BOX, USA), followed by membrane transfer using the iBlot™ 2 Dry Blotting System (Invitrogen #IB21001, USA), and immunoblotting was performed using the iBind™ Western System (Invitrogen #SLF1000, USA). Detection in the Western blot utilized the BeyoECL Moon (Ultra-sensitive ECL chemiluminescent reagent kit, (Beyotime #P0018FS, Shanghai, China). The validated eluted proteins were sent to BGI (Beijing Genomics Institute, China) for mass spectrometry analysis using the Q-Exactive HF X mass spectrometer. Following peptide segment analysis, the results were compared against the protein database of *N. benthamiana* (https://www.uniprot.org/uniprotkb?query=(taxonomy_id:4085), accessed on 20 February 2023), and excluding the protein IDs displayed in the control.

## 5. Conclusions

Pst-DPBB has a signal peptide at its N-terminus, and this signal peptide has been experimentally verified to possess secretion functionality. Pst-DPBB is induced to be highly expressed early during the interaction between the wheat stripe rust fungus and wheat, and it may localize to the cytoplasm, nucleus, and apoplast, participating in multiple biological processes. Pst-DPBB can inhibit INF1-induced cell death in tobacco and DC3000-induced cell death in barley, demonstrating its function in negatively regulating plant immune responses. Pst-DPBB can suppress callose accumulation in barley and negatively regulate the flg22-induced PTI response, indicating its role in the PTI response of plants. Fifteen candidate interaction targets of Pst-DPBB were identified using Co-IP-MS/MS technology.

## Figures and Tables

**Table 1:** The candidate interactors of Pst-DPBB in *Nicotiana benthamiana*.

Protein ID	Description
A0A1S5WM37	Papain-like cysteine proteinase 6
D2Y4N3	Ankyrin repeat-containing protein
E3W9P7	Kirola-like; putative PR-10 type pathogenesis-related protein
A0A1U7W9S1	Glucose-6-phosphate isomerase
A0A7T7D836	ATP synthase subunit beta, chloroplastic
A0A1J6JK69	Cysteine synthase
A0A1U7W460	Cysteine proteinase 3
A0A1S3YBR4	Proteasome subunit beta type-1-like isoform X2
A0A1S3ZJA2	Cinnamoyl-CoA reductase 1-like
A0A1S3XFR3	Macrophage migration inhibitory factor homolog
A0A1J6K253	Putative carboxylesterase 17
A0A1S3Y5P8	Serpin-ZX-like
A0A1S3XHC6	Cinnamoyl-CoA reductase 2-like
A0A1S4BTY9	Sulfurtransferase
A0A1U7WQU4	Epimerase family protein SDR39U1 isoform X2

## Data Availability

The data presented in this study are available on request from the corresponding author.

## Supplementary Materials

The following supporting information can be downloaded at: https://www.mdpi.com/article/10.3390/plants14030435/s1, Figure S1: Pst-DPBB coding sequence alignment; Figure S2: Detection of GFP and the ΔSP-Pst-DPBB-GFP fusion protein using GFP Trap Agarose immunoprecipitation; Figure S3: The sequence alignment of cysteine proteinase; Table S1: Pst-DPBB structural homologs and annotations in *Puccinia striiformis*; Table S2: Pst-DPBB structural homologs in PDB Database; Table S3: Specific primers used in this study.
