# Supplementary material for: A Puccinia striiformis f. sp. tritici Effector with DPBB Domain Suppresses Wheat Defense
Source: Plants (Basel). 2025 Feb 2;14(3):435. doi: 10.3390/plants14030435 (PMC11820871; doi:10.3390/plants14030435)
Supplement: Supplementary file 1 [file plants-14-00435-s001.zip › supplementary figures.pdf]

```
CYR32        ATGCATTCCAGAGCTCTTGTCGCTTCAATCCTTTTCTCATCCAACATCGCTTGGGCATCAACGATCGGTAGTAGAGCAGATTTCAATCATCATCCTCGCG   100
PST-11-281   ATGCATTCCAGAGCTCTTGTCGCTTCAATCCTTTTCTCATCCAACATCGCTTGGGCATCAACGATCGGTAGTAGAGCAGATTTCAATCATCATCCTCGCG   100
PST-38S102   ATGCATTCCAGAGCTCTTGTCGCTTCAATCCTTTTCTCATCCAACATCGCTTGGGCATCAACGATCGGTAGTAGAGCAGATTTCAATCATCATCCTCGCG   100
PST-93-210   ATGCATTCCAGAGCTCTTGTCGCTTCAATCCTTTTCTCATCCAACATCGCTTGGGCATCAACGATCGGTAGTAGAGCAGATTTCAATCATCATCCTCGCG   100
PST-K        ATGCATTCCAGAGCTCTTGTCGCTTCAATCCTTTTCTCATCCAACATCGCTTGGGCATCAACGATCGGTAGTAGAGCAGATTTCAATCATCATCCTCGCG   100
PST-78       ATGCATTCCAGAGCTCTTGTCGCTTCAATCCTTTTCTCATCCAACATCGCTTGGGCATCAACGATCGGTAGTAGAGCAGATTTCAATCATCATCCTCGCG   100
PST-Yr9      ATGCATTCCAGAGCTCTTGTCGCTTCAATCCTTTTCTCATCCAACATCGCTTGGGCATCAACGATCGGTAGTAGAGCAGATTTCAATCATCATCCTCGCG   100
Consensus    atgcattccagagctcttgtcgcttcaatccttttctcatccaacatcgcttgggcatcaacgatcggtagtagagcagatttcaatcatcatcctcgcg

CYR32        GGCTCACTTCCGGAGAGGCGCGGTACTATGAACTCGACACCGGGGCGCAAACCACTTGTGGTGGATTTCATAAGAGTACAGAGATGATCTGCGCTCTTGG   200
PST-11-281   GGCTCACTTCCGGAGAGGCGCGGTACTATGAACTCGACACCGGGGCGCAAACCACTTGTGGTGGATTTCATAAGAGTACAGAGATGATCTGCGCTCTTGG   200
PST-38S102   GGCTCACTTCCGGAGAGGCGCGGTACTATGAACTCGACACCGGGGCGCAAACCACTTGTGGTGGATTTCATAAGAGTACAGAGATGATCTGCGCTCTTGG   200
PST-93-210   GGCTCACTTCCGGAGAGGCGCGGTACTATGAACTCGACACCGGGGCGCAAACCACTTGTGGTGGATTTCATAAGAGTACAGAGATGATCTGCGCTCTTGG   200
PST-K        GGCTCACTTCCGGAGAGGCGCGGTACTATGAACTCGACACCGGGGCGCAAACCACTTGTGGTGGATTTCATAAGAGTACAGAGATGATCTGCGCTCTTGG   200
PST-78       GGCTCACTTCCGGAGAGGCGCGGTACTATGAACTCGACACCGGGGCGCAAACCACTTGTGGTGGATTTCATAAGAGTACAGAGATGATCTGCGCTCTTGG   200
PST-Yr9      GGCTCACTTCCGGAGAGGCGCGGTACTATGAACTCGACACCGGGGCGCAAACCACTTGTGGTGGATTTCATAAGAGTACAGAGATGATCTGCGCTCTTGG   200
Consensus    ggctcacttccggagaggcgcggtactatgaactcgacaccggggcgcaaaccacttgtggtggatttcataagagtacagagatgatctgcgctcttgg

CYR32        TATATCCGCTTTTGGCACAGGCGAGCACTGTGGAAAGTCTCTAGTAATCCATCACGGCGGTAAACAAGTGACGTGTATGGTGGATGACAAATGTCAATCG   300
PST-11-281   TATATCCGCTTTTGGCACAGGCGAGCACTGTGGAAAGTCTCTAGTAATCCATCACGGCGGTAAACAAGTGACGTGTATGGTGGATGATAAATGTCAATCG   300
PST-38S102   TATATCCGCTTTTGGCACAGGCGAGCACTGTGGAAAGTCTCTAGTAATCCATCACGGCGGTAAACAAGTGACGTGTATGGTGGATGACAAATGTCAATCG   300
PST-93-210   TATATCCGCTTTTGGCACAGGCGAGCACTGTGGAAAGTCTCTAGTAATCCATCACGGCGGTAAACAAGTGACGTGTATGGTGGATGACAAATGTCAATCG   300
PST-K        TATATCCGCTTTTGGCACAGGCGAGCACTGTGGAAAGTCTCTAGTAATCCATCACGGCGGTAAACAAGTGACGTGTATGGTGGATGACAAATGTCAATCG   300
PST-78       TATATCCGCTTTTGGCACAGGCGAGCACTGTGGAAAGTCTCTAGTAATCCATCACGGCGGTAAACAAGTGACGTGTATGGTGGATGATAAATGTCAATCG   300
PST-Yr9      TATATCCGCTTTTGGCACAGGCGAGCACTGTGGAAAGTCTCTAGTAATCCATCACGGCGGTAAACAAGTGACGTGTATGGTGGATGATAAATGTCAATCG   300
Consensus    tatatccgcttttggcacaggcgagcactgtggaaagtctctagtaatccatcacggcggtaaacaagtgacgtgtatggtggatga aaatgtcaatcg

CYR32        TGTACGGCCAATTCCCTGGATGTATCTCCCGCCGTCTTCCAAGCGCTTGCACCGTTATCTGAGGGAGTACTAAAAGTCGACTGGAACTTCGTCTG        395
PST-11-281   TGTACGGCCAATTCCCTGGATGTATCTCCCGCCGTCTTCCAAGCGCTTGCACCGTTATCTGAGGGAGTACTAAAAGTCGACTGGAACTTCGTCTG        395
PST-38S102   TGTACGGCCAATTCCCTGGATGTATCTCCCGCCGTCTTCCAAGCGCTTGCACCGTTATCTGAGGGAGTACTAAAAGTCGACTGGAACTTCGTCTG        395
PST-93-210   TGTACGGCCAATTCCCTGGATGTATCTCCCGCCGTCTTCCAAGCGCTTGCACCGTTATCTGAGGGAGTACTAAAAGTCGACTGGAACTTCGTCTG        395
PST-K        TGTACGGCCAATTCCCTGGATGTATCTCCCGCCGTCTTCCAAGCGCTTGCACCGTTATCTGAGGGAGTACTAAAAGTCGACTGGAACTTCGTCTG        395
PST-78       TGTACGGCCAATTCCCTGGATGTATCTCCCGCCGTCTTCCAAGCGCTTGCACCGTTATCTGAGGGAGTACTAAAAGTCGACTGGAACTTCGTCTG        395
PST-Yr9      TGTACGGCCAATTCCCTGGATGTATCTCCCGCCGTCTTCCAAGCGCTTGCACCGTTATCTGAGGGAGTACTAAAAGTCGACTGGAACTTCGTCTG        395
Consensus    tgtacggccaattccctggatgtatctcccgccgtcttccaagcgcttgcaccgttatctgagggagtactaaaagtcgactggaacttcgtctg
```

Figure S1. Pst-DPBB coding sequence alignment. Coding sequence alignment of Pst-DPBB (KNE92489.1) across seven *Puccinia striiformis* f. sp. *tritici* (Pst) isolates: PST-78 (GCA_001191645.1), CYR32 (GCA_000474995.1), PST-38S102 (GCA_001936605.2), PST-Yr9 (GCA_002008965.1), PST-K (GCA_002008935.1), PST-11-281 (GCA_004194325.1), and PST-93-210 (GCA_002920065.1). The alignment reveals a SNP at position 288 among the races.

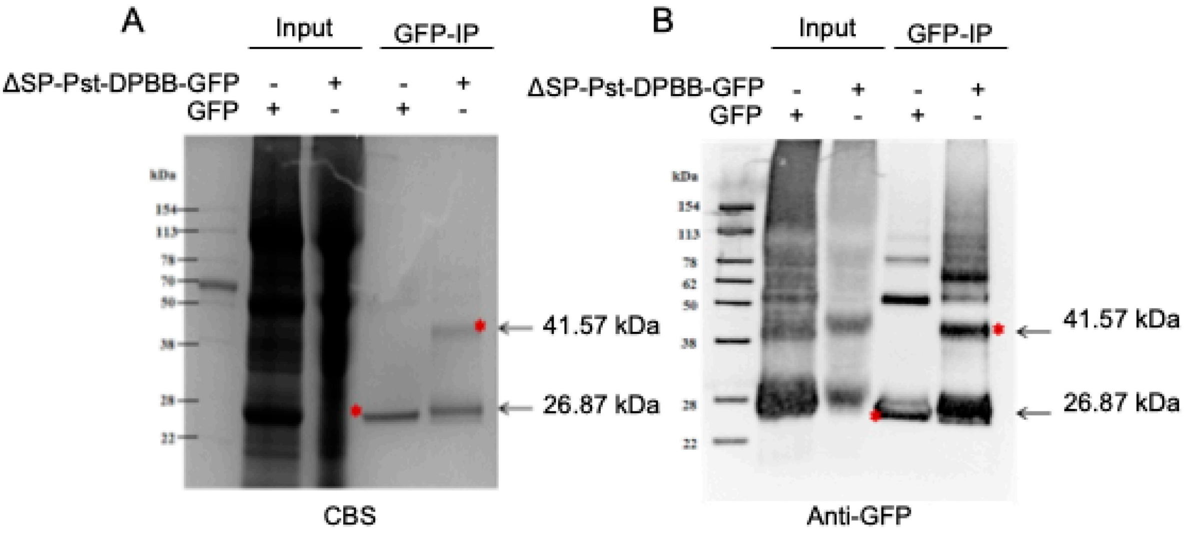

Figure S2. Detection of GFP and the ΔSP-Pst-DPBB-GFP fusion protein using GFP Trap Agarose immunoprecipitation. Total proteins were extracted from the leaves of *N. benthamiana* at 10 days post-infiltration (dpi) with *Agrobacterium* carrying either pTRBO-GFP (26.87 kDa) or pTRBO-ΔSP-Pst-DPBB-GFP (41.57 kDa). The proteins eluted from the GFP Trap Agarose were analyzed using Coomassie blue staining (**A**) and western blotting (**B**) with an anti-GFP antibody.

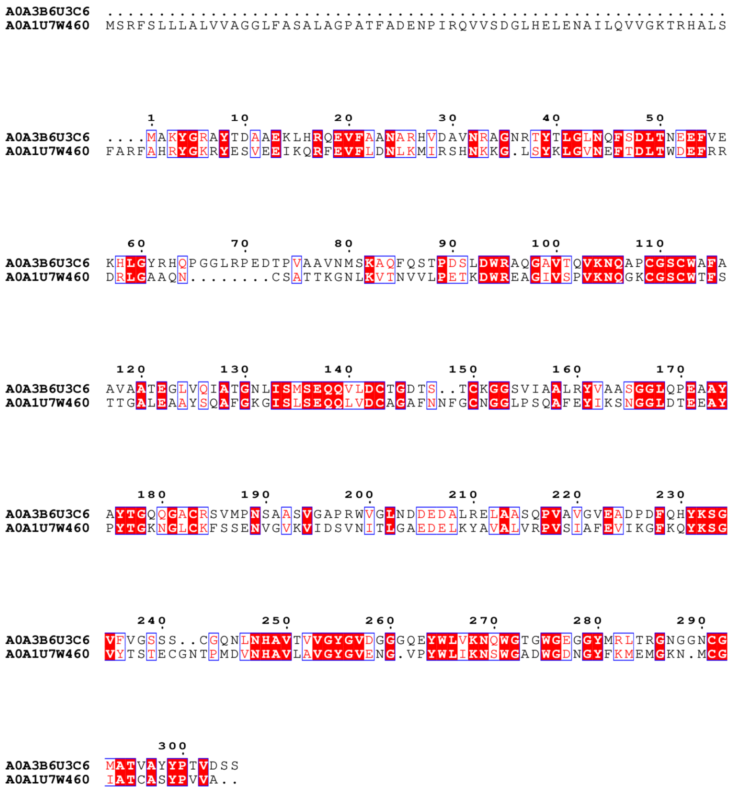

Figure S3. The sequence alignment of cysteine proteinase. It illustrates the similarity between the wheat cysteine proteinase (A0A3B6U3C6) identified as interacting with PST-DPBB through AlphaFold3 (ipTM = 0.66) and *N. benthamiana* cysteine proteinase 3 (A0A1U7W460) identified via Co-IP MS/MS. The two sequences share 30% identity, suggesting potential conservation of function.
