# Supplementary material for: A Puccinia striiformis f. sp. tritici Effector with DPBB Domain Suppresses Wheat Defense
Source: Plants (Basel). 2025 Feb 2;14(3):435. doi: 10.3390/plants14030435 (PMC11820871; doi:10.3390/plants14030435)
Supplement: Supplementary file 1 [file plants-14-00435-s001.zip › Table S3.pdf]

Table S3. Specific primers used in this study

	Primers	Sequence (5'-3')
Construction of recombinant vector for subcellular localization	pTRBO-G Fwd	atggctagcaaaggagaagaac
	pTRBO-G Rev	ttaattaatgacaagaacacgaactgag
	SP-Pst-DPBB Fwd	cttgtcattaattaaatgcattccagagctcttgtcgc
	SP-Pst-DPBB Rev	tcctttgctagccatgacgaagttccagtcgac
Construction of recombinant vector for signal peptide validation	Pst-DPBB$^{1-57}$ Fwd	cgtgttcttgtcattaattaaatgcattccagagctcttgtcgc
	Pst-DPBB$^{1-57}$ Rev	accgggacacttgaatgcccaagcgatgttg
	ΔSP-Pst-SCR1 Fwd	ttcaagtgtcccggtttg
Verification for vector construction	pJL-TRBO-G Left	tccatctcagttcgtgttcttg
	pJL-TRBO-G Right	accacgtgtgattacggaca
Construction of recombinant vector for BSMV-HIGS	Pst_DPBB(210) Fwd	ttctaaggaagggccgtaatccagagctcttgtcgcttc
	Pst_DPBB(210) Rev	ttaaccaccaccaccgggccaaaagcggatataccaag
qRT-PCR	qRT-Pst-DPBB Fwd	tttggcacaggcgagcactg
	qRT-Pst-DPBB Rev	ttggaagacggcgggagatacat
	qRT-PstEF-1α Fwd	ttcgccgtccgtgatatgagacaa
	qRT-PstEF-1α Rev	atgcgtatcatggtggtggagtga
	qRT-NbEF1α Fwd	ctacctcaagaaggttggatac
	qRT-NbEF1α Rev	aacatcctgaagtggaagac
	qRT-NbCYP71D20 Fwd	accgcaccatgtccttagag
	qRT-NbCYP71D20 Rev	cttgccccttgagtacttgc
	qRT-NbPR2 Fwd	gggctgttaatttgcagtatcc
	qRT-NbPR2 Rev	ggtttataacatcttggtctgatgg
	qRT-NbWRKY12 Fwd	ctcatcagctagttcatttgatgc
	qRT-NbWRKY12 Rev	agctcggtctttgttctaaaagc
	qRT-NbAct-qRT Fwd	gttgctatacaagctgttctctcg
	qRT-NbAct-qRT Rev	gtcaagacgaagaatgacatgtgg
	qRT-NbPR1a Fwd	cgaccaggtagcagcctatg
	qRT-NbPR1a Rev	tctcaacagccttagcagcc
